# Knowledge, Perceptions, and Practices of Primary Care Physicians on HIV and PrEP: Challenges and Principles of PrEP Use

**DOI:** 10.3390/healthcare13080854

**Published:** 2025-04-09

**Authors:** Aleksandra Kozieł, Igor Domański, Natalia Kuderska, Bartosz Szetela, Aleksandra Szymczak, Brygida Knysz

**Affiliations:** 1 Department of Internal Medicine and Diabetology with Subdivision of Endocrinology, Independent Public Regional Hospital in Szczecin, 71-455 Szczecin, Poland; 2 Clinical Department of Diabetology, Hypertension and Internal Diseases, Wroclaw Medical University, 50-556 Wroclaw, Poland; igor.domanski@umw.edu.pl; 3 1st Psychiatric Department, Lower Silesian Centre of Mental Health, 50-226 Wroclaw, Poland; natkuderska@gmail.com; 4 Department of Infectious Diseases, Liver Disease and Acquired Immune Deficiencies, Wroclaw Medical University, 50-367 Wroclaw, Polandaleksandra.szymczak@umw.edu.pl (A.S.); brygida.knysz@umw.edu.pl (B.K.); 5 All Saint’s Clinic, Wrocławskie Centrum Zdrowia SP ZOZ, 50-136 Wroclaw, Poland

**Keywords:** HIV, Poland, PrEP, knowledge, primary healthcare (PHC)

## Abstract

**Background/Objective**: HIV remains a significant public health issue in Poland, with many diagnoses occurring at advanced stages due to the limited access to diagnostic tools in primary healthcare (PHC). General practitioners are crucial for early detection, but barriers such as the absence of rapid testing in PHC settings hinder a timely diagnosis. This study evaluates primary care physicians’ knowledge of HIV diagnostics and pre-exposure prophylaxis (PrEP), focusing on their role in improving prevention and early detection. **Material and Methods**: This study used anonymous surveys distributed online and on paper to physicians in randomly selected primary care facilities across four Polish voivodeships. The data were analysed statistically to assess their knowledge, attitudes, and practices related to HIV diagnostics, rapid testing, and PrEP. **Results**: A total of 100 surveys were collected. A total of 83% of the physicians reported recommending HIV tests, although 17% had never done so, mainly among family medicine specialists. A total of 88% were aware of Voluntary Counselling and Testing Centres (VCTs), but 99% had never performed rapid HIV tests in their offices. Physicians with shorter professional experience (less than 10 years) demonstrated a significantly higher awareness of PrEP compared to that in those with longer experience (Fisher’s test *p* = 0.35). **Conclusions**: Primary care physicians play a crucial role in HIV prevention, but limited access to diagnostic tools and systemic support hampers their effectiveness. Targeted education and a comprehensive program for STI and HIV prevention are needed to improve prevention efforts and early detection.

## 1. Introduction

Human Immunodeficiency Virus (HIV) infection remains a significant public health issue in Poland. Despite increasing social awareness, including within key populations, a high incidence of new infections persists. In recent years, there has even been a recorded rise in the number of cases [1]. This increase is largely associated with the growing availability and improvement of HIV testing methods.

In Poland, a high proportion of patients are diagnosed at an advanced stage of HIV infection (so-called late-presenters) [2], defined as a CD4 lymphocyte count of <350 cells/µL or the presence of indicator diseases [3]. Early diagnosis and the prompt initiation of antiretroviral therapy play a pivotal role in effective HIV epidemic control [4]. Primary healthcare (PHC) serves a particularly crucial yet often undervalued function in the diagnostic process [5,6,7,8,9]. General practitioners (GPs), including family physicians, are frequently the first specialists with the opportunity to identify symptoms suggestive of HIV infection. Their role in initiating diagnostic testing and referring patients to the appropriate diagnostic pathway is critical for accelerating the recognition and treatment process. Unfortunately, the standard diagnostic panel available in PHC in Poland does not include screening for HIV. Primary care facilities (PCFs) do not receive direct funding for reimbursement for HIV testing. HIV testing is typically covered under public health programs when performed in specific settings, such as infectious disease clinics or Voluntary Counselling and Testing Centres (VCTs). Consequently, the diagnostic process is often prolonged, as patients must obtain a referral to an infectious diseases clinic, visit VCTs for anonymous testing (available mainly in the largest cities), or undergo private testing. Regrettably, HIV infection may remain undiagnosed for many years, with patients being referred to various specialists unable to diagnose the condition within their fields [10,11,12].

In Poland, pre-exposure prophylaxis (PrEP) is available—a method involving the use of antiretroviral drugs by HIV-negative individuals to reduce their risk of acquiring an infection [13,14]. In 2015, the European Centre for Disease Prevention and Control (ECDC) issued a recommendation that European Union (EU) countries should consider integrating PrEP into their existing HIV prevention programs for individuals at the highest risk of infection [15]. In the same year, the World Health Organization (WHO) released similar recommendations for all countries globally [16]. The updated guidelines from 2022, titled “Global Health Sector Strategies on HIV, Viral Hepatitis, and Sexually Transmitted Infections for the Period 2022–2030”, recognise the widespread adoption of PrEP as a key strategy in combating the epidemic [17,18]. However, both in Poland and across Europe, PrEP remains a relatively unknown and underutilised prevention method, particularly among physicians not specialising in infectious diseases [19]. A 2019 study based on data from the European MSM Internet Survey estimated that approximately 17.4% of men who have sex with men (MSM) in the EU—around 500,000 individuals—who were highly willing to use PrEP did not have access to it [20]. Increasing the availability and utilisation of PrEP thus remains a critical public health priority.

The aim of this study was to assess the knowledge of primary care physicians regarding symptoms suggestive of HIV infection, its diagnostics, and their familiarity with PrEP. Additionally, this article outlines the principles of PrEP use that can be applied in everyday medical practice.

## 2. Materials and Methods

This study was conducted using anonymous, self-designed surveys targeting physicians working in primary healthcare facilities. The surveys were written in Polish. Some were distributed in paper form directly at PHC centres, while others were made available to the physicians online. The healthcare facilities were selected randomly. This study primarily focused on the Lower Silesian Voivodeship, particularly Wroclaw, as well as the Lubusz Voivodeship, mainly Zielona Góra, and the Greater Poland and West Pomeranian Voivodeships. The collected data, both in paper and electronic formats, were subsequently subjected to a statistical analysis.

The survey examined several key areas related to the practices and awareness of primary care physicians in the diagnostics and prevention of HIV. It assessed the respondents’ professional experience and specialisation. This study analysed whether the physicians recommended HIV testing and the factors that motivated them to do so, as well as their knowledge of the operation and locations of Voluntary Counselling and Testing Centres (VCTs). The survey also investigated whether the physicians performed rapid cassette tests for HIV and identified potential barriers to their use. Additionally, it explored their willingness to conduct such tests if they were readily available, as well as the reasons for their potential reluctance to perform them. The final section of the survey focused on pre-exposure prophylaxis (PrEP), examining the physicians’ knowledge of this prevention method, their experience in prescribing antiretroviral drugs, and the reasons why they might avoid this practice.

All of the variables included in the analysis were categorical. To describe the dataset, frequency tables, bar charts, and expected frequency tables were used. To test the hypothesis of independence between pairs of variables, chi-square tests were applied when all of the expected cell counts were equal to or greater than 5. In cases where this assumption was not met, Fisher’s exact test was used instead.

To account for multiple comparisons and control the probability of Type I errors, the Benjamini–Hochberg correction was applied to each set of hypotheses. This method was chosen to maintain the highest possible statistical power.

The representativeness of the study group regarding the length of their professional experience was analysed, showing that most of the experience length classes were proportionally represented compared to the population-based expectations. The data were collected from databases made available by the Polish Central Statistical Office and the Polish General Medical Council for the year 2023. Confidence intervals for the proportions were calculated using the “binom.confint” function from the R (4.4.2) package “binom” (1.1-1.1) for a sample size of n = 100.

For the class of <1 year of experience, the observed proportion was 7%, with a confidence interval of 3.43–13.75%, which did not include the expected proportion of 2%. This indicates the overrepresentation of this group in the study sample. For the remaining classes of experience length, namely 1–5 years, 6–10 years, and >10 years, the observed proportions fell within the determined confidence intervals, reflecting consistency with population data.

## 3. Results

A total of 100 surveys were collected in this study, including 79 in paper form and 21 electronically. For the surveys distributed directly, all of the respondents (100%) completed them. However, since the electronic survey was distributed across several PHC facilities, it was challenging to determine the precise number of individuals who had access to the form compared to the number who actually responded.

**Figure 1:**
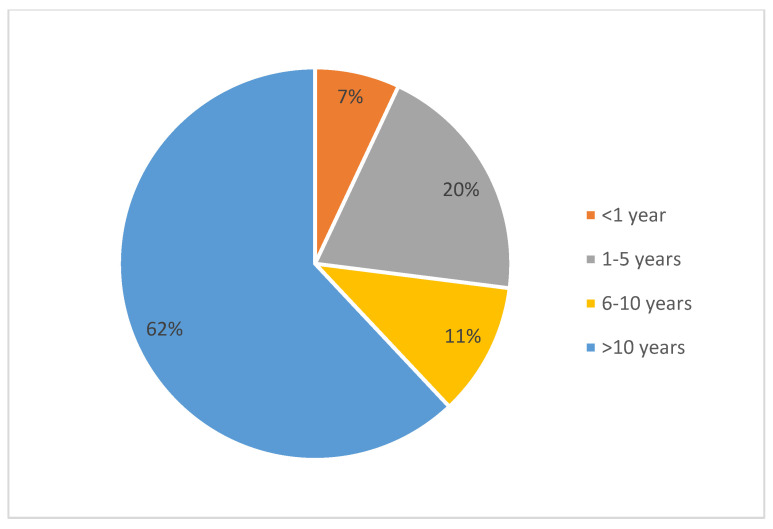
Distribution of respondents by length of service in primary care.

**Figure 2:**
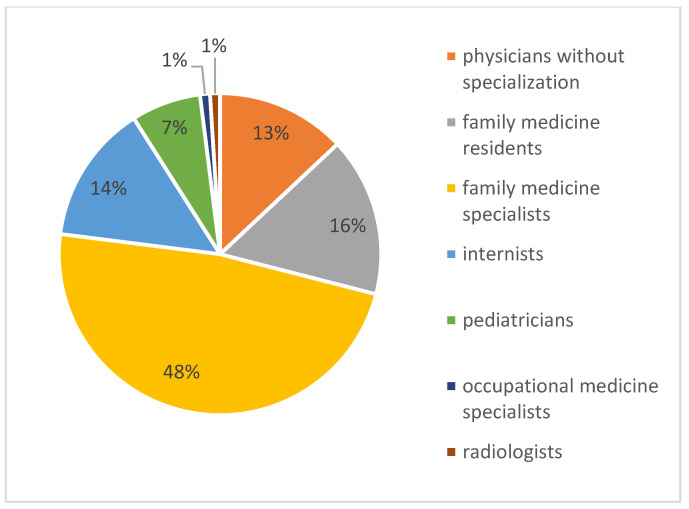
Percentage share of physician groups in the survey study.

The largest group in terms of professional experience in PHC consisted of individuals with over 10 years of experience, accounting for 62% of the respondents. Regarding their specialisation, the most numerous groups were family medicine specialists, who made up 48% of all of the respondents (Figure 1 and Figure 2).

**Figure 3:**
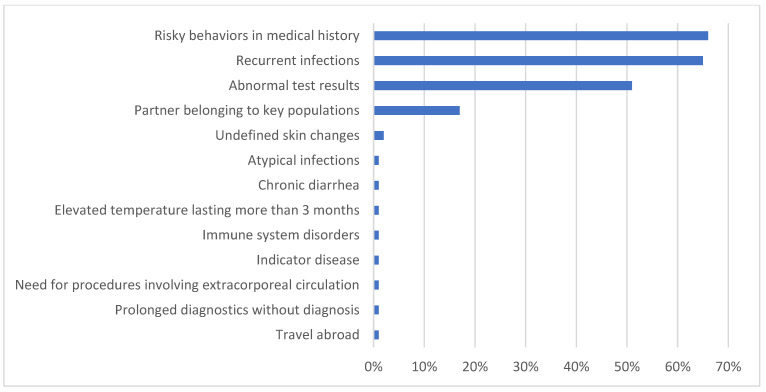
Reasons for ordering HIV tests in primary care physicians.

The vast majority of the respondents (83%) reported recommending an HIV test to a patient at least once during their practice. Their reasons for ordering HIV tests are presented in Figure 3.

Only 17% of the physicians had never recommended an HIV test to a patient. This group was dominated by family medicine specialists and family medicine residents, with each representing 29%. Most of the physicians who had never recommended an HIV test had over 10 years of professional experience (59%); however, this correlation was not statistically significant (Fisher’s test *p* = 0.119).

A total of 88% of the respondents were aware of the existence of Voluntary Counselling and Testing Centres (VCTs), where patients can undergo testing for HIV, HCV, and syphilis anonymously and free of charge. Among those unaware of VCTs, 75% had over 10 years of professional experience, with family medicine specialists making up the largest proportion (42%). However, neither correlation was statistically significant (Fisher’s test: *p* = 0.527 and *p* = 0.165, respectively).

The majority of the physicians who were aware of VCTs recommended their use to patients (78%) and were aware of the location of their nearest VCT (74%).

Almost all of the physicians (99%) reported never having performed a rapid cassette HIV test in their office, primarily due to the unavailability of this tool (90%). The significant majority of the physicians (73%) stated that they would use the option of performing HIV testing in their PHC office if it were available. Those who did not consider this option (14%) or were undecided (11%) cited reasons such as limited time during visits, concerns about handling patients’ blood during finger pricks, doubts about the test’s reliability, fear of patients’ reactions to being offered the test, insufficient knowledge about the tests, and a preference for VCTs, where other infections can also be ruled out.

**Figure 4:**
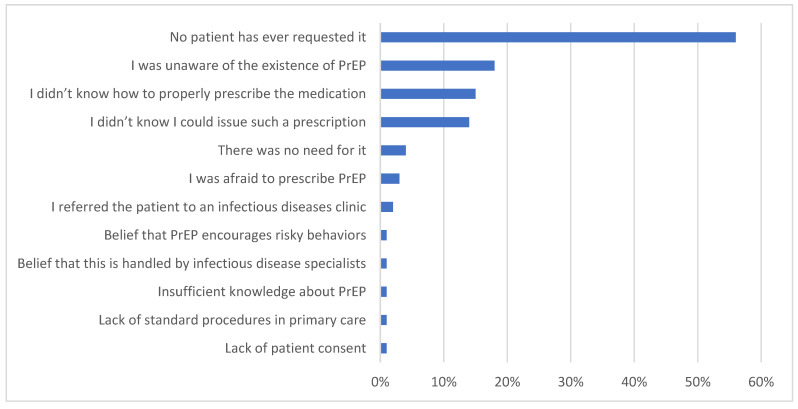
Reasons why physicians chose not to prescribe PrEP.

Most of the physicians (70%) were familiar with pre-exposure prophylaxis (PrEP), but 94% of them had never prescribed it to a patient. The reasons for the physicians’ reluctance to prescribe PrEP are presented in Figure 4.

The majority of those unfamiliar with PrEP (30%) were individuals with longer professional experience, exceeding 10 years (83%). Conversely, among the physicians with less professional experience—of less than 1 year or 1 to 5 years—the awareness of PrEP was significantly higher, at 86% and 90%, respectively. The physicians in primary healthcare with less experience (<1 year and 1–5 years) demonstrated a higher level of knowledge about PrEP compared to that in those with more experience (>10 years) (Fisher’s test *p* = 0.35). However, this knowledge did not correlate with the frequency of prescribing PrEP—only 6% of the physicians prescribed PrEP.

## 4. Discussion

Among the surveyed physicians, the vast majority (83%) have recommended an HIV test to a patient at least once in their practice. This is a very positive sign, indicating that doctors are aware of the risks associated with HIV infection and can recognise symptoms that may suggest this disease. While this result is promising, it may reflect some bias within the studied group. To determine whether this is truly a reliable and representative trend, a study involving a larger group of physicians would be necessary.

In Poland, a high rate of late diagnoses is observed. A lack of awareness of HIV symptoms among doctors may contribute to this issue [2]. In a study conducted between 2004 and 2006, over 40% of 235 final-year medical students at the Bialystok Medical University claimed that there were no symptoms that could prompt a physician to suspect HIV infection, despite 50% of the students rating their knowledge about HIV and AIDS as good or very good [21]. In a survey conducted in 2017 in Poland among 42 physicians undergoing a residency in family medicine, only 5 doctors (11.9%) knew with certainty that there were symptoms based which a physician could reasonably suspect an HIV infection [22]. In studies conducted in Germany between 2009 and 2013, the symptoms most commonly overlooked by physicians included thrombocytopenia, oral candidiasis, unexplained weight loss, shingles, and cervical dysplasia or cervical cancer in women [23].

Most of the physicians (72%) expressed willingness to perform a rapid HIV cassette test in the primary care setting; however, due to the unavailability of such tests, they had no opportunity to use them. The reasons cited for reluctance to perform testing included a lack of time, concerns about handling blood, and doubts about the reliability of the tests. Similar concerns were reported in a 2018 review analysing the knowledge of HIV prevention and diagnostics among family physicians across Europe [24]. This review highlighted that a lack of time was the most significant barrier to performing rapid HIV testing during consultations. Physicians argued that the pre-test discussion could be too complex and time-consuming. Another critical limitation mentioned was their apprehension about interpreting the results and communicating a potential HIV diagnosis [24]. A study conducted in the UK found that appropriate training of medical staff could help integrate HIV testing as a routine component of patient care [24,25].

Despite their concerns, the significant majority of the respondents demonstrated willingness to offer rapid tests to patients. Providing physicians with access to this tool in their practice could greatly enhance the chances of them detecting HIV infections among patients in primary care settings. Introducing anti-HIV antibody testing into the reimbursed diagnostics for primary care physicians could also significantly expedite the diagnostic process. Family doctors, through their long-term care of patients, establish enduring relationships. In cases of suspected HIV infection, a diagnostic proposal from a trusted physician may increase a patient’s willingness to take appropriate action [26,27]. Additionally, it is crucial for family physicians to actively initiate conversations about sexually transmitted infections. A survey conducted between 2014 and 2015 among patients of a public medical facility in the USA revealed that the majority of the respondents—65.8%—would have liked their physician to offer them an HIV test. Among the participants initially unwilling for their doctor to propose an HIV test, over half (59.3%) stated that they would agree to testing if their doctor recommended it. This study showed that a physician’s recommendation was the most common reason that patients were willing to undergo HIV testing [28]. Similar findings were reported in a 2012 U.S. survey, where one-third of the respondents who had ever been tested for HIV indicated that their decision was influenced by a physician’s recommendation. Conversely, among those who had never been tested, about one-third cited the lack of a doctor’s recommendation as the primary reason [29]. In a study conducted among New York residents, individuals who received a physician’s recommendation of an HIV test were ten times more likely to undergo testing compared to those who did not receive such a recommendation [30]. A 2018 review examining the HIV testing practices among family physicians in Europe found that offering tests without an explicit request by the patient or an urgent diagnostic need made family doctors feel uncomfortable. They expressed concerns about potential patient reactions [24]. Overcoming the perception of HIV infection as a taboo topic and fostering open conversations with patients are crucial to improving this country’s epidemiological situation.

The physicians who did not recommend HIV testing to their patients were predominantly those with more professional experience (>10 years). A lack of knowledge about what and where Voluntary Counselling and Testing Centres (VCTs) were, as well as a limited awareness of pre-exposure prophylaxis (PrEP), were also more common in this group of more experienced physicians. Similar conclusions were presented in a 2010 study involving over 200 family practice clinics in the United Kingdom. Younger physicians were more likely to order tests for sexually transmitted infections and were more open to discussing sexuality with their patients compared to their more experienced counterparts [31]. Comparable findings were observed in an Italian study from 2015, which surveyed physicians working with key populations and individuals living with HIV. Most of the doctors who were either unfamiliar with PrEP or opposed to its use were over 45 years old [32]. Younger doctors are more familiar with the symptoms that may indicate HIV infection, as well as the modern methods of prevention and diagnostics. This is likely due to the increased emphasis on education about HIV and AIDS in medical curricula in recent years. These results highlight the need for the continuous re-education of senior physicians and updates to their knowledge about HIV, particularly regarding the latest standards in diagnostics and prevention.

Although 70% of the surveyed physicians were familiar with PrEP, only one reported ever prescribing it to a patient. By comparison, in a 2017 study conducted in the U.S. among 280 family physicians, 75% were aware of PrEP; however, only one-third had discussed it with their patients, and just 17% had prescribed it. This study demonstrated that the level of knowledge about PrEP is a key factor influencing physicians’ confidence and their ability to engage in discussions about PrEP and make prescribing decisions [33]. In a German study from 2019, it was shown that among HIV specialists, 30% proactively encouraged at-risk patients to use PrEP. In contrast, no cases of proactive PrEP counselling were observed among non-HIV-specialist physicians [19].

It is essential to note that patients may be unaware of the availability of this preventive option [34]. Physicians in primary care settings should proactively inquire about potential infection risks, especially among key populations. A lack of questions from a patient does not absolve a physician from the responsibility of providing information. In this study, 14% of the respondents indicated they did not know they could prescribe pre-exposure prophylaxis. According to the law, any physician can prescribe PrEP [35,36]. However, in practice, it is most commonly prescribed by infectious disease specialists and physicians focusing on sexually transmitted infections. This requires knowledge of their indications and the risks faced by the patient.

A significant barrier to PrEP’s accessibility is the lack of reimbursement, meaning that patients must cover not only the cost of the medication but also the expenses of monitoring for potential side effects. However, it is worth noting that the treatment for individuals living with HIV in Poland is publicly funded, and the costs are substantial. For antiretroviral therapy, the annual cost per person is approximately 30,000 PLN, depending on the treatment regimen [37]. The average age of patients with newly diagnosed HIV is around 33 years [2], and individuals living with HIV typically have a lifespan only slightly shorter than that of uninfected individuals [38,39]. Following an HIV diagnosis, a patient may live an additional 40–50 years on average, which translates to a total lifetime treatment cost of approximately 1.6 million PLN per person. These are significant expenditures that the state could avoid by promoting effective diagnostic and preventive methods.

## 5. Recommendations for Primary Care Physicians

In everyday practice in primary healthcare facilities, it is important to consider so-called indicator diseases, such as bacterial, recurrent community-acquired pneumonia, or a fever of unknown origin. Attention should be paid to skin changes such as extensive shingles, rashes, chronic ulcers of an HSV aetiology, or mucosal and skin candidiasis [40,41,42,43]. Patients should also be referred for testing when infectious mononucleosis with a severe course is suspected, as this may manifest as acute retroviral syndrome [44]. Testing for HIV infection should be recommended for individuals with abnormal laboratory results without an apparent cause [40]. Physicians should also pay attention to pregnant women and ask whether they have undergone HIV testing during pregnancy. Pregnant women should be educated that the proper testing is conducted twice—once in the first trimester and again in the third trimester of pregnancy [45].

PrEP should be used in adults at risk of HIV infection, including, among others, individuals engaging in unprotected sexual activity with partners of an unknown serostatus or with people living with HIV who are untreated or ineffectively treated. Prophylaxis should also be applied to individuals engaging in sexual activity under the influence of psychoactive substances, intravenous drug users, sex workers, or those who explicitly request the initiation of PrEP [46]. Before initiating prophylaxis, HIV and HBV infections should be excluded and serum creatinine levels should be measured. Additionally, other sexually transmitted infections (STIs), including syphilis, HCV, chlamydia, and gonorrhoea, should be ruled out [46]. Physicians should also discuss the principles of taking PrEP during the visit and the risks of other sexually transmitted infections (Table 1).

In Poland, the following drug combinations are used: tenofovir disoproxil fumarate (TDF) [300 mg] or tenofovir alafenamide (TAF) [25 mg] combined with emtricitabine (FTC) [200 mg], taken as one tablet daily on a continuous basis. Studies indicate that protection for anal and vaginal intercourse is achieved after 7 days of use, whereas for intravenous injections, it is achieved after 20 days. For cisgender men and transgender individuals assigned male at birth, on-demand use is also possible—two TDF/FTC tablets 2–24 h before intercourse, followed by one tablet 24 h after the initial dose and one tablet 48 h after the initial dose [46]. For transgender individuals, the PrEP dosing should be tailored individually based on the stage of their transition, their medication use, and the type of sexual activities engaged in. Women can only use PrEP continuously [46].

When PrEP is used correctly, its efficacy in preventing HIV infection during sexual contact is as high as 99%. A lower protection efficacy is observed among individuals who use intravenous drugs (74%) and patients using PrEP on demand (approximately 86%) [14]. It is extremely important to explain to patients that the effectiveness of the medication depends on adherence [47,48].

These medications, when used over a longer period, require monitoring. The first follow-up is recommended after one month, with subsequent follow-ups every three months. During these visits, HIV infection should be ruled out, a medical history concerning sexually transmitted infections should be conducted, and, if necessary, diagnostic procedures should be initiated. Additionally, serum creatinine levels should be measured.

**Figure 5:**
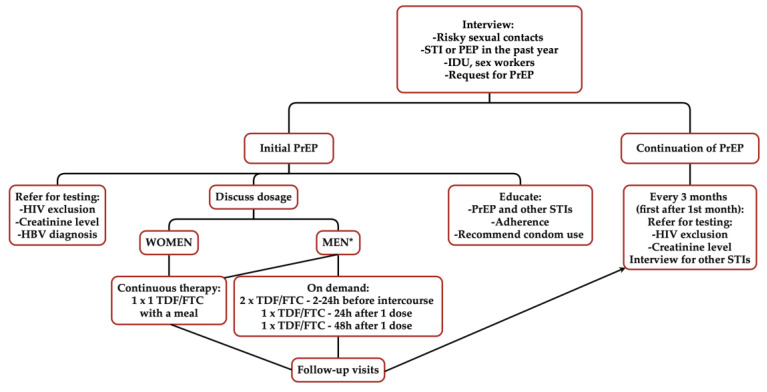
Workflow diagram; own elaboration. * Cis men and transgender people assigned male at birth who do not take estradiol; STI—sexually transmitted infection, PEP—post-exposure prophylaxis, IDU—intravenous drug users, PrEP—pre-exposure prophylaxis, HIV—human immunodeficiency virus, HBV—hepatitis B virus, TDF—tenofovir disoproxil fumarate, FCT—emtricitabine.

PrEP, like any other medication, may cause side effects. Most of these are mild and resolve quickly, primarily involving gastrointestinal symptoms such as abdominal pain and nausea. A relatively rare side effect is impaired kidney function, which is why kidney function is assessed at every follow-up visit [46] (Table 1, Figure 5).

Importantly, when prescribing PrEP, it should be emphasised that it only protects against HIV infection and does not prevent other sexually transmitted infections. It is crucial to explain to patients that PrEP should not be used as the sole preventive method and that condoms are essential. PrEP serves as an additional layer of protection, but mechanical protection should remain the foundation of prevention.

## 6. Limitations

A limitation of this study is the overrepresentation of physicians with the shortest professional experience (<1 year), which may affect the generalisability of the results to the entire population. Furthermore, due to the lack of detailed data on the specialisation structure of the physicians working in primary care in Poland, it was not possible to assess the representativeness of the sample based on this criterion.

This study was conducted primarily in the Lower Silesian and Lubusz Voivodeships, specifically in Wroclaw and Zielona Góra. Both cities have Voluntary Counselling and Testing (VCT) centres, which may have contributed to the greater knowledge about these centres among the respondents. Additionally, the results may depend on local educational programs, the availability of training, and support from healthcare institutions, limiting the comparability of the findings with other regions that have varying levels of access to diagnostic and educational resources.

The paper-based surveys were distributed in person, which may have contributed to a higher response rate. However, the number of respondents to the online survey is difficult to determine due to the inability to precisely estimate the number of individuals who received the survey. The survey-based methodology also limited the ability to expand the respondents’ knowledge about HIV and PrEP.

Most of the correlations in this study came out as statistically insignificant due the study group being too small. Studies on a larger group are needed to be able to estimate trends in the population of doctors in Poland.

## 7. Conclusions

Primary care physicians are key to HIV prevention and diagnosis, yet their role is hindered by limited access to rapid tests, a lack of systemic support (e.g., no PrEP reimbursement), and insufficient training. Although many are willing to engage in testing and prevention, knowledge gaps—especially among senior doctors—remain a major barrier. In Poland, there is a need for a comprehensive STI prevention program involving primary care, specialists, and clinics, with clear diagnostic guidelines, better tools, and regular training. Enhanced support from the Ministry of Health and integration into national health policy would strengthen education, improve early detection, and support effective HIV and STI control.

Further research in this field is necessary, involving a larger study group.

## Figures and Tables

**Table 1:** Guidelines for patient care before and during pre-exposure prophylaxis (PrEP) use.

Before Prescribing PrEP:	During PrEP Use (1-Month Follow-Up, Subsequent Visits Every 3 Months):
The following should be applied: - Exclude an HIV infection; - Measure serum creatinine levels; - Conduct HBV diagnostics; - Discuss the medication dosing, emphasise the importance of adherence, and highlight that in addition to using PrEP, it is crucial to use condoms.	The following should be applied: - Exclude an HIV infection; - Conduct a medical history focusing on other sexually transmitted infections; - Measure creatinine levels.
Additionally, it is recommended to pursue the following: - Administer vaccinations for HBV, HAV, and HPV; - Diagnose other sexually transmitted infections (e.g., syphilis, HCV, chlamydia, gonorrhoea); - Perform additional tests, such as a complete blood count and urinalysis.	
During each visit, it is recommended to provide education, emphasise the importance of condom use, and stress adherence.	

## Data Availability

The data presented in this study are only available on request from the corresponding author due to privacy, legal, or ethical reasons.
